# RECRUITMENT OF SOMMA PARTICIPANTS: TECHNIQUES TO ENROLL OLDER ADULTS IN A COMPLEX STUDY

**DOI:** 10.1093/geroni/igac059.832

**Published:** 2022-12-20

**Authors:** Anne Newman, Peggy Cawthon, Theresa Mau, Stephen Kritchevsky, Russell T Hepple, Paul Coen, Bret Goodpaster, Steven Cummings

**Affiliations:** University of Pittsburgh, Pittsburgh, Pennsylvania, United States; California Pacific Medical Center Research Institute, San Francisco, California, United States; California Pacific Medical Center, San Francisco, California, United States; Wake Forest School of Medicine, Winston-Salem, North Carolina, United States; University of Florida, Gainesville, Florida, United States; AdventHealth, Orlando, Florida, United States; AdventHealth, Orlando, Florida, United States; San Francisco Coordinating Center, San Francisco, California, United States

## Abstract

We screened approximately 4200 individuals to enroll 881 including 41% men and 59% women with a mean age =73.4 ± 5SD years; 13% were Black, 85% White and 2% of other race and ethnic groups. We will add 80 individuals aged 30-69 years. Exclusions included unstable medical conditions and inability to walk 400 meters(m). The median baseline 400m gait speed at usual pace was 1.05 ± 0.18SD m/s. Techniques to recruit and enroll a diverse population for this complex project included the use of in-person and video-conference information sessions to explain the complicated study to interested adults and to screen potential participants as needed The COVID pandemic created numerous challenges; while SOMMA was able to meet its recruitment goals, the enrollment period lasted 33 months (instead of the originally planned 18 months). Attendance at follow-up in-person and phone-based assessments is excellent.

